# Staphylococcal Biofilm Exopolysaccharide Protects against Caenorhabditis elegans Immune Defenses

**DOI:** 10.1371/journal.ppat.0030057

**Published:** 2007-04-20

**Authors:** Jakob Begun, Jessica M Gaiani, Holger Rohde, Dietrich Mack, Stephen B Calderwood, Frederick M Ausubel, Costi D Sifri

**Affiliations:** 1 Department of Molecular Biology, Massachusetts General Hospital, Boston, Massachusetts, United States of America; 2 Department of Genetics, Harvard Medical School, Boston, Massachusetts, United States of America; 3 Division of Infectious Diseases and International Health, University of Virginia Health System, Charlottesville, Virginia, United States of America; 4 Institut für Medizinische Mikrobiologie, Virologie und Hygiene, Universitätsklinikum Hamburg-Eppendorf, Hamburg, Germany; 5 Medical Microbiology and Infectious Diseases, The School of Medicine, University of Wales Swansea, Swansea, United Kingdom; 6 Division of Infectious Diseases, Massachusetts General Hospital, Boston Massachusetts, United States of America; 7 Department of Microbiology and Molecular Genetics, Harvard Medical School, Boston, Massachusetts, United States of America; Stanford University, United States of America

## Abstract

Staphylococcus epidermidis and Staphylococcus aureus are leading causes of hospital-acquired infections that have become increasingly difficult to treat due to the prevalence of antibiotic resistance in these organisms. The ability of staphylococci to produce biofilm is an important virulence mechanism that allows bacteria both to adhere to living and artificial surfaces and to resist host immune factors and antibiotics. Here, we show that the *icaADBC* locus, which synthesizes the biofilm-associated polysaccharide intercellular adhesin (PIA) in staphylococci, is required for the formation of a lethal S. epidermidis infection in the intestine of the model nematode Caenorhabditis elegans. Susceptibility to S. epidermidis infection is influenced by mutation of the C. elegans PMK-1 p38 mitogen-activated protein (MAP) kinase or DAF-2 insulin-signaling pathways. Loss of PIA production abrogates nematocidal activity and leads to reduced bacterial accumulation in the C. elegans intestine, while overexpression of the *icaADBC* locus in S. aureus augments virulence towards nematodes. PIA-producing S. epidermidis has a significant survival advantage over *ica*-deficient S. epidermidis within the intestinal tract of wild-type *C. elegans,* but not in immunocompromised nematodes harboring a loss-of-function mutation in the p38 MAP kinase pathway gene *sek-1*. Moreover, *sek-1* and *pmk-1* mutants are equally sensitive to wild-type and *icaADBC*-deficient S. epidermidis. These results suggest that biofilm exopolysaccharide enhances virulence by playing an immunoprotective role during colonization of the C. elegans intestine. These studies demonstrate that C. elegans can serve as a simple animal model for studying host–pathogen interactions involving staphylococcal biofilm exopolysaccharide and suggest that the protective activity of biofilm matrix represents an ancient conserved function for resisting predation.

## Author Summary

Biofilm is an agglomeration of microbes bound together by a slimy matrix composed of excreted proteins and polysaccharide polymers. Most bacteria in the environment reside in biofilms, as do 80% or more of those causing human infections, according to some estimates. During infection, biofilm matrix acts as a safe haven, protecting bacterial cells from antibiotics, immune cells, and antimicrobial factors. In this report, we demonstrate that the ability of Staphylococcus epidermidis to produce a lethal infection within the intestinal tract of the roundworm Caenorhabditis elegans depends on the S. epidermidis intercellular adhesion *(ica)* locus, which is responsible for the synthesis of the principal exopolysaccharide of staphylococcal biofilm, polysaccharide intercellular adhesin (PIA). Using a collection of bacterial and nematode mutants, we show that PIA promotes infection by working against protective immune factors controlled by the C. elegans SEK-1 PMK-1 p38 mitogen-activated protein kinase pathway. In addition to providing further evidence for the immunoprotective function of the biofilm polymer PIA, these results show that *C. elegans* can be used in a simple, live animal model for the study of host–pathogen interactions involving biofilm matrix.

## Introduction

Staphylococci are a predominant cause of hospital-acquired infections, particularly those associated with implanted medical devices and catheters. The ability of staphylococci, particularly *Staphylococcus epidermidis,* to form biofilm on biotic and abiotic surfaces appears to be critical for the establishment of these infections and to contribute to their persistence by protecting S. epidermidis from antibiotics and host defenses [1,2]. Bacterial biofilm is composed of multilayered cell clusters encased in an exopolysaccharide matrix. The biofilm matrix of S. epidermidis consists mainly of partially deacetylated β-1,6-linked polymeric *N*-acetyl glucosamine (PNAG), commonly referred to as polysaccharide intercellular adhesin (PIA) [3]. PIA mediates intercellular adhesion essential for biofilm accumulation, and also has a role in primary attachment to certain hydrophilic abiotic polymer surfaces [3–7]. The S. epidermidis intercellular adhesion locus *(ica),* consisting of the biosynthetic operon *icaADBC* and the regulatory gene *icaR* [5,8,9], is required for PIA biosynthesis as well as for biofilm formation in vitro and in several animal models of S. epidermidis infection [6,10,11]. In addition to its role in surface and cell-to-cell adherence, biofilm also appears to provide an immunoprotective function, reducing the ability of phagocytes to engulf S. epidermidis and protecting S. epidermidis from antimicrobial peptides [12,13]. Epidemiologic studies have shown that the presence of the *ica* locus is associated with pathogenic strains of S. epidermidis [14–16].

In contrast to *S. epidermidis,* biofilm formation by Staphylococcus aureus appears to be more robust in vivo than in vitro, which may be a consequence of phenotypic switching or other regulatory differences [17,18]. Nevertheless, some S. aureus isolates produce biofilm on glucose- and/or sucrose-supplemented rich media or under anaerobic conditions, and most S. aureus strains contain the *icaADBC* operon [19–23]. Isolation of a spontaneous S. aureus mutant that produces copious amounts of biofilm in vitro, MN8m, led to the characterization of a 5-nucleotide regulatory sequence within the *icaADBC* promoter that normally acts to repress transcription [24]. Deletion of this region, as in MN8m, results in increased *icaADBC* transcription and enhanced biofilm formation [24]. Thus, the *icaADBC* operon may normally be repressed in S. aureus in vitro, which explains why relatively few S. aureus strains form biofilm under standard laboratory conditions. Nevertheless, the *ica* locus contributes to S. aureus virulence in several animal models of invasive S. aureus disease [25].

While loss of the *icaADBC* operon generally leads to a reduction of pathogenicity and biofilm formation of S. epidermidis and S. aureus in vivo, complete loss of staphylococcal virulence is generally not observed [10,25,26]. Moreover, the *ica* locus does not appear to contribute to S. epidermidis and S. aureus biofilm formation in guinea pig and mouse tissue cage infection models, perhaps due to the binding of staphylococci to host matrix molecules that coat the implanted cages [27,28].

Recently, the nematode Caenorhabditis elegans has proven to be a facile model for studying the interaction between microbial pathogens and host factors and examining the contribution of specific gene products to virulence and immunity [29,30]. A key feature of the C. elegans pathogenicity models is that many Gram-positive virulence factors previously identified to be important for mammalian pathogenesis have also been shown to play important roles in the infectious process in C. elegans [31–34]. Recently, biofilm formation has also been implicated as a virulence factor in C. elegans infection models for the Gram-negative pathogens Yersinia pestis and *Yersinia pseudotuberculosis,* which inhibit C. elegans growth by forming an obstructive mass over the pharyngeal opening of the nematode [35,36].

The C. elegans pathogenesis model system has also been used to study the nematode innate immune system [30,37]. In contrast to mammals and insects, the Toll signaling pathway may not play a significant role in C. elegans immune defenses. C. elegans lacks known MyD88 and Rel/NFκB homologs, and partial loss-of-function mutation of *tol-1,* the single Toll-like receptor (TLR) gene in *C. elegans,* does not alter susceptibility to several pathogens [38]. However, three evolutionarily conserved signaling pathways have been found to impact nematode immunity to human pathogens: the DAF-2 insulin-signaling pathway [39], the TGF-β pathway [40,41], and the p38 mitogen-activated protein (MAP) kinase pathway [42].

The p38 MAP kinase pathway is composed of the MAP kinase kinase kinase NSY-1, the MAP kinase kinase SEK-1, and the MAP kinase PMK-1, which are homologous to the mammalian proteins ASK-1, MKK3/6, and p38 MAP kinase, respectively [42]. *sek-1* and *nsy-1* mutants display enhanced susceptibility to a range of pathogens, including the Gram-positive bacteria Enterococcus faecalis and S. aureus [32,42]. Similar involvement of the p38 MAP kinase pathway in mammalian immune responses has led to the hypothesis that the pathway is an ancestral immune signal system that predates the development of TLR-dependent immune signaling pathways [30].

Key components of the insulin-signaling pathway include the insulin receptor gene *daf-2* and the phosphatidylinositol 3-kinase catalytic subunit gene *age-1*. DAF-2 and AGE-1 act through PDK-1 kinase and AKT-family kinases to phosphorylate, and thereby impede, nuclear translocation of the forkhead transcription factor DAF-16/FOXO [43–46]. Loss-of-function mutation of *daf-2* or *age-1* increases lifespan, entry into dauer diapause, and resistance to oxidative stress and bacterial infection, all in a *daf-16*-dependent manner [39,47–49]. Recent data suggest that the p38 MAP kinase pathway and the DAF-2 insulin-signaling pathway function in parallel in the innate immune response [50].

Here, we use a C. elegans–S. epidermidis pathogenesis system to study the role of biofilm exopolysaccharide in bacterial pathogenesis. We show that S. epidermidis causes a lethal infection of the C. elegans intestinal tract and that disruption of the *icaADBC* locus prevents long-term bacterial colonization, reduces total bacterial accumulation, and greatly diminishes nematode killing. Furthermore, overexpression of the *S. aureus icaADBC* locus in S. aureus enhances virulence. An important experimental advantage of the C. elegans pathogenicity models is that genetic analysis can be carried out in both the pathogen and host simultaneously, a process we have termed “interactive genetic analysis” [29]. Taking advantage of both pathogen and host mutants, we show here that wild-type and *ica*-deficient S. epidermidis strains kill at similar rates when they infect nematodes with defects in p38 MAP kinase signaling and accumulate to equivalent levels during infection of these immunocompromised hosts. These results demonstrate that the *C. elegans–S. epidermidis* pathogenesis system can be used as a live animal model for studying the role of biofilm exopolysaccharide in bacterial pathogenesis from the perspective of both the pathogen and the host.

## Results

### S. epidermidis Kills C. elegans through an Active Infectious Process

**Figure 1:**
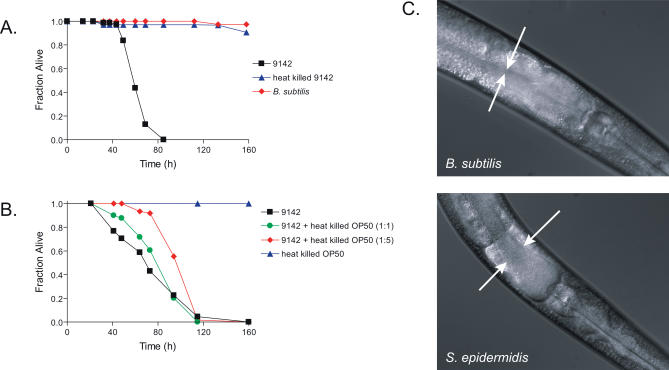
S. epidermidis Kills C. elegans and Causes Intestinal Distension (A) *C. elegans* killing assays on lawns of live S. epidermidis 9142 (squares), heat-killed S. epidermidis 9142 (triangles), or B. subtilis strain RL2244 (diamonds). (B) Survival of C. elegans exposed to live S. epidermidis 9142 (squares), admixtures of live S. epidermidis 9142 and heat-killed E. coli OP50 at a ratio of 1:1 (circles) or 1:5 (diamonds), or heat-killed E. coli OP50 alone (triangles). (C) N2 C. elegans were exposed to B. subtilis RL2244 or S. epidermidis 9142 for 24 h and then visualized by Nomarski differential contrast microscopy. Arrows demarcate the intestinal tract lumen. Magnification, ×40.

When feeding on their normal laboratory food source Escherichia coli strain OP50 or on other relatively benign bacteria such as Bacillus subtilis strain PY79, C. elegans has a lifespan of approximately 2 wk [39,51]. In contrast, C. elegans exhibit a considerably shorter lifespan when feeding on a variety of human pathogenic bacteria [29,52]. As is the case when C. elegans are fed several other pathogens, nematodes fed certain laboratory and clinical strains of S. epidermidis or other coagulase-negative staphylococci die over the course of 3–5 d (Figures 1A and S1).

Several observations indicate that the *C. elegans–S. epidermidis* interaction involves an infectious process. First, heat-killed S. epidermidis does not kill C. elegans (Figure 1A), and S. epidermidis culture supernatants do not lead to significant worm mortality (unpublished data). Furthermore, nematodes fed a mixture of live S. epidermidis and heat-killed OP50 (an innocuous food source to ensure adequate nutritional content) at an equal or 1:5 ratio, respectively, die within several days, although at a slightly reduced rate as compared to S. epidermidis alone (Figure 1B). Taken together, the data in Figure 1A and 1B demonstrate that the decreased lifespan requires live bacteria rather than stable secreted toxins and is not due simply to S. epidermidis being a poor source of nutrition.

The second line of evidence for an infectious process is that although S. epidermidis–mediated killing of C. elegans is accompanied by the matricidal hatching of eggs within the uterus of the hermaphrodite mother (similar to other pathogens that have been investigated [32,53]), this is not the main cause of death since C. elegans males, which do not produce embryos, are also killed by S. epidermidis (unpublished data).

Third, similar to other microbial pathogens that kill *C. elegans,* large numbers of intact S. epidermidis cocci accumulate within the intestinal tract of nematodes during the course of infection, leading to significant distension of the intestinal lumen compared to nematodes feeding on B. subtilis (Figure 1C). It should be noted, however, that accumulation of bacteria within the nematode intestinal tract is not sufficient for killing: aerobically-cultured Enterococcus faecium accumulates to high titers within the digestive tract but is not appreciably harmful to wild-type nematodes [31].

**Figure 2:**
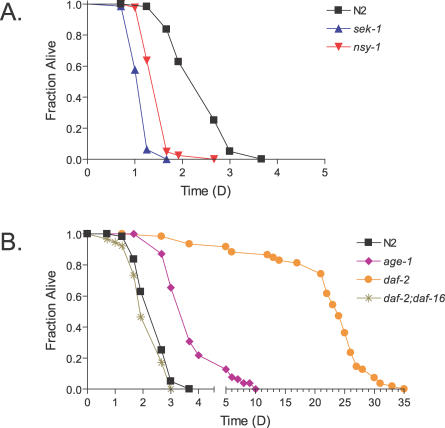
C. elegans Mutants with Altered Immune Function Show Differential Sensitivity to S. epidermidis (A) Survival of wild-type N2 C. elegans (squares) compared to survival of the immunocompromised, p38 MAP kinase pathway mutants *sek-1(ag1)* (triangles, *p* < 0.0001) or *nsy-1(ag3)* (inverted triangles, *p* < 0.0001) when exposed to S. epidermidis 9142. (B) Survival of N2 animals (squares) compared to survival of the pathogen-resistant, insulin signaling pathway mutants *age-1(hx546)* (diamonds, *p* < 0.0001) or *daf-2(e1370)* (circles, *p* < 0.0001) when exposed to S. epidermidis 9142. Survival of *daf-2(e1370);daf-16(mgDf47)* mutants (asterisks) demonstrates that *daf-16(mgDf47)* suppresses *daf-2(e1370)* enhanced pathogen resistance.

A final reason to conclude that the *C. elegans–S. epidermidis* model involves an infectious process is that the altered susceptibility exhibited by C. elegans innate immunity-related mutants when fed S. epidermidis is comparable to the observed phenotypes of these mutants exposed to other pathogens that infect C. elegans. *nsy-1* and *sek-1* encode the MAPKKK and MAPKK, respectively, of a conserved p38 MAP kinase signaling pathway in *C. elegans,* and mutations in these genes result in enhanced susceptibility to a variety of pathogens, including S. aureus [32]. When *nsy-1(ag3)* and *sek-1(ag1)* mutant nematodes are fed *S. epidermidis,* they display significantly enhanced susceptibility to infection, as shown in Figure 2A (*p* < 0.0001).

Conversely, C. elegans genes *daf-2* and *age-1* encode components of an insulin-signaling pathway, and mutations in these genes increase nematode longevity on innocuous bacteria as well as greatly increase resistance to infection by Gram-positive pathogens [39]. As shown in Figure 2B, *daf-2(e1370)* and *age-1(hx546)* mutants are remarkably less susceptible to S. epidermidis–mediated killing (*p* < 0.0001). Similar to other pathogens that have been tested [39], the extension of lifespan on S. epidermidis is disproportionate to that observed during exposure to innocuous bacteria. For example, *daf-2(e1370)* mutants exposed to S. epidermidis have a 10-fold extension in lifespan compared to wild-type nematodes (Figure 2B), whereas the average lifespan of *daf-2(e1370)* mutants are, at most, doubled when exposed to the non-pathogens OP50 and PY79 [39]. DAF-2 and AGE-1 negatively regulate the forkhead transcription factor DAF-16/FOXO, and, as expected, survival of *daf-2(e1370);daf16(mgDf47)* animals is comparable to wild-type N2 nematodes (*p >* 0.05), demonstrating that DAF-2–mediated pathogen resistance requires DAF-16 signaling (Figure 2B). Survival of *daf-16(mgDf47)* mutants exposed to S. epidermidis is comparable to N2 nematodes (unpublished data), similar to results published previously for S. aureus [39].

### *S. epidermidis ica* Locus Is Required for Colonization and Killing of C. elegans

**Figure 3:**
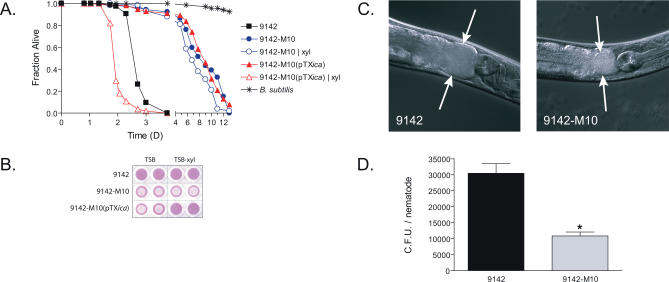
S. epidermidis Virulence in the C. elegans Infection Model Depends on *icaADBC* (A) Survival of C. elegans infected with S. epidermidis strain 9142-M10 (circles), which contains a transposon insertion in the *icaA* gene, and the complemented strain 9142-M10(pTX*ica*) (triangles), which carries the *icaADBC* operon driven by the xylose-inducible P*xylA* promoter, compared to wild-type S. epidermidis 9142 (squares) and B. subtilis (asterisks). Survival assays were performed under standard conditions (closed symbols) or using plates supplemented with 2% xylose (xyl, open symbols). (B) Biofilm formation of S. epidermidis 9142, 9142-M10, and 9142-M10(pTX*ica*) on polystyrene. Attachment to polystyrene 96-well flat bottom microtiter plate was performed as described in the Materials and Methods. Strains were grown in TS broth without supplementation (TSB) or supplemented with 2% xylose (TSB-xyl). (C) Nomarski micrograph of C. elegans after feeding for 24 h on S. epidermidis 9142 or 9142-M10. Arrows demarcate the intestinal tract lumen. Magnification, ×40. (D) Quantification of intestinal bacteria obtained by disruption of worms after 24 h of feeding on either S. epidermidis 9142 or 9142-M10. Values represent the mean of five samples with approximately eight worms per sample ± standard error of the mean (SEM). The asterisk indicates a significant difference (*p* < 0.001).

Biofilm is a major virulence factor of S. epidermidis in mammalian pathogenesis that promotes adherence to artificial surfaces and protects S. epidermidis from antibiotics and immune effectors. To investigate the contribution of biofilm exopolysaccharide to S. epidermidis–mediated killing of *C. elegans,* nematodes were fed S. epidermidis strain 9142-M10, which produces no detectable PIA and is completely biofilm-deficient as a result of a Tn*917* insertion in the *icaA* gene [4,26,54]. As shown in Figure 3A, 9142-M10 exhibits significantly decreased virulence relative to the wild-type isogenic parental strain 9142 (*p* < 0.0001). To confirm that the reduced virulence of 9142-M10 was due to interruption of *icaA,* plasmid pTX*icaADBC,* which contains the *icaADBC* operon driven by the P*xylA* xylose-inducible promoter [55,56], was introduced into 9142-M10 (see Materials and Methods). The complemented mutant, 9142-M10(pTX*ica*), was able to form as robust a biofilm on polystyrene as the wild-type parental strain under 2% xylose-inducing conditions, but was as biofilm-deficient as 9142-M10 when grown without xylose supplementation (Figure 3B). Similarly, the nematocidal activity of 9142-M10(pTX*ica*) exceeded that of 9142 when grown in the presence of 2% xylose, but was as attenuated as 9142-M10 when grown without supplemental xylose. Survival of C. elegans grown on 9142 or 9142-M10 was not meaningfully changed with xylose supplementation (Figure 2A and unpublished data).

Previous work has shown that growth inhibition of C. elegans by Y. pestis and Y. pseudotuberculosis also depends on biofilm production, and specifically on the *hmsHFRS* locus, a homolog of *icaADBC* [35]. In these infections, an obstructive biofilm plug forms over the mouth of the nematode, starving the animals. However, microscopic examination of nematodes feeding on S. epidermidis showed no sign of obstructive masses or bacterial adherence to the cuticle (unpublished data). To further test whether bacterial adhesion to the surface of nematodes was a factor in S. epidermidis infection of nematodes, we tested the susceptibility of a set of C. elegans mutants with altered cuticle structure. The C. elegans mutants *srf-2, srf-3,* and *srf-5* display wild-type sensitivity to S. epidermidis (unpublished data). In contrast, these mutants have been shown to be resistant to biofilm-mediated cuticle infection by Y. pseudotuberculosis [36,57]. These data suggest that S. epidermidis virulence does not depend on its ability to adhere to the external surfaces of worms.

To further examine the relationship between bacterial virulence and PIA production, nematodes feeding on wild-type and PIA-deficient S. epidermidis strains were examined microscopically. As shown in Figure 3C, both wild-type S. epidermidis 9142 and the *ica* mutant 9142-M10 accumulate in the C. elegans intestine; however, worms feeding on 9142-M10 appear to have less intestinal distension. To determine if the reduced distension is a result of decreased colonization of the intestinal tract, the number of live intestinal bacteria was quantified. As shown in Figure 3D, C. elegans feeding on 9142-M10 have significantly fewer colony-forming units (C.F.U.) in their intestines than worms feeding on wild-type bacteria (*p* < 0.001).

### The *ica* Locus Confers a Competitive Survival Advantage to S. epidermidis during Infection of C. elegans

To date, two distinct mechanisms of nematode killing associated with intestinal tract colonization have been described: transient infection and persistent infection [29]. In the latter case, brief exposure to some pathogens, such as E. faecalis and *Salmonella enterica,* leads to lethal infection that is associated with bacterial retention and proliferation in the C. elegans intestinal tract [31,58]. In contrast, *S. aureus,* typical of those pathogens that cause a transient infection, is completely expelled from the intestinal tract within 2 h of nematodes being transferred to another food source [32]. Consequently, continuous exposure to S. aureus is necessary to achieve maximal worm killing [32]. Since both S. epidermidis and S. aureus colonize the nematode intestinal tract and kill worms, we examined whether the mechanism of worm killing by S. epidermidis was similar to that of S. aureus.

**Figure 4:**
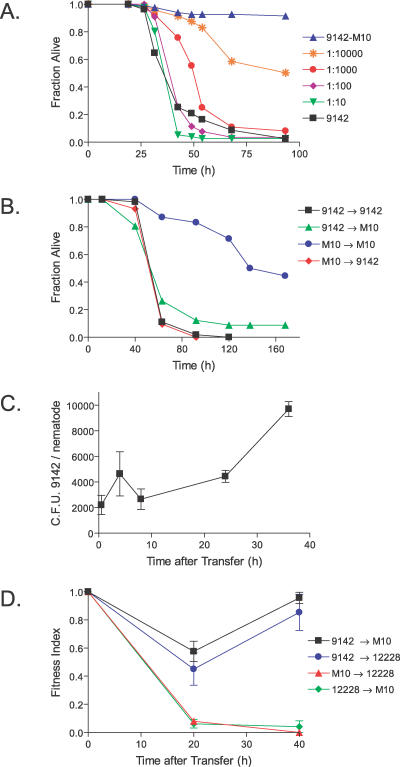
The Fitness of Intraluminal S. epidermidis during Colonization of C. elegans Is *icaADBC*-Dependent (A) Survival of nematodes feeding on mixed lawns of S. epidermidis 9142 and 9142-M10 in the ratios of 9142:9142-M10 indicated. (B) C. elegans exposed to S. epidermidis 9142 for 12 h and then transferred to S. epidermidis 9142-M10 (triangles) die with similar kinetics to worms transferred from 9142 to 9142 (squares) or 9142-M10 to 9142 (diamonds) and much more rapidly than control worms transferred from 9142-M10 to 9142-M10 (circles). (C) Intestinal proliferation of S. epidermidis 9142 over time in C. elegans feeding on 9142 for 12 h and transferred to 9142-M10. Values represent the mean of three samples with approximately ten worms per sample ± SEM. (D) Nematodes exposed to S. epidermidis 9142 for 12 h and transferred either to 9142-M10 (squares) or a second *ica*-deficient S. epidermidis strain ATCC 12228 (circles) retain a high proportion of 9142 in their intestinal tracts, whereas those exposed first to the *ica*-deficient 9142-M10 and transferred to *ica*-deficient ATCC 12228 (triangles), or vice versa (diamonds), do not retain the initial bacteria in their digestive tracts. Fitness Index is defined as: (pulse S. epidermidis strain C.F.U.) / (pulse S. epidermidis strain C.F.U. + chase S. epidermidis strain C.F.U.). Values represent the mean of three samples with approximately ten worms per sample ± SEM.

First, we evaluated the relative virulence of wild-type and PIA-deficient S. epidermidis by exposing nematodes to lawns of 9142 diluted in 9142-M10. As shown in Figure 4A, significant nematode killing occurred with exposure to mixed lawns of 9142 and 9142-M10 in ratios as low as 1:10,000. That a relatively small amount of wild-type S. epidermidis has significant nematocidal activity suggests that PIA-producing S. epidermidis may preferentially colonize the C. elegans intestinal tract compared to *ica*-deficient 9142-M10. Alternatively, it could be hypothesized that killing by PIA-producing S. epidermidis is not bacterial density–dependent.

To further examine the mechanism of S. epidermidis infection, nematodes were fed wild-type S. epidermidis 9142 for 12 h and then transferred to plates containing *ica*-deficient 9142-M10. As shown in Figure 4B, transferred nematodes die with similar kinetics as worms fed exclusively wild-type bacteria, suggesting that the *ica* mutant 9142-M10 was not capable of rescuing the worms and that durable colonization is established within 12 h of exposure to *S. epidermidis,* a time at which there is no observed nematode mortality. In contrast, 9142-M10 is capable of being an effective “rescue” food for worms that have been fed *S. aureus;* that is, when *S. aureus*–fed C. elegans were transferred to S. epidermidis 9142-M10, the worms survived significantly longer than worms that remained on the S. aureus plates (unpublished data).

Taken together, these data suggest that a small inoculum of or brief exposure to S. epidermidis may be sufficient to establish a long lasting, lethal infection. To verify this, nematodes were exposed to 9142 and then transferred to 9142-M10, as above, and intestinal bacteria were recovered by mechanical disruption and quantified at various time points. Recovered bacteria were serially diluted on tryptic soy (TS) agar containing Congo Red dye to distinguish wild-type and biofilm exopolysaccharide–negative colonies [16]. As shown in Figure 4C, wild-type S. epidermidis persists and accumulates over time within the intestinal tract of worms transferred to 9142-M10.

To determine whether S. epidermidis persists in the intestinal tract irrespective of the rescue food source, S. epidermidis–fed nematodes were transferred to E. faecium E007. As we have previously observed for S. aureus–fed nematodes [32], C. elegans exposed to S. epidermidis 9142 were rescued from lethal infection when transferred to E007 (unpublished data). The fact that E. faecium was an effective rescue food for worms infected with *S. epidermidis,* whereas an *ica S. epidermidis* mutant did not rescue worms previously infected with wild-type *S. epidermidis,* led us to hypothesize that the competitive advantage exhibited by 9142 during mixed infections with 9142-M10 is the result of biofilm exopolysaccharide. To test this, we first exposed nematodes to wild-type S. epidermidis 9142 and then transferred them to either the 9142-M10 or to ATCC 12228 [59], an *ica*-deficient S. epidermidis reference strain, which is not pathogenic to worms, as shown in Figure S1. Prior to transfer and 20 and 40 h after transfer, nematodes were collected and intestinal tract bacteria were quantified by serial dilution plating on TS agar plate containing Congo Red dye, as above [16]. As shown in Figure 4D, wild-type bacteria persist in the intestinal tract of worms transferred to lawns of PIA-deficient bacteria, becoming the vast majority of the population 40 h after transfer, despite the fact that the nematodes were feeding exclusively on biofilm exopolysaccharide–deficient strains. As was observed in Figure 4C, the absolute number of wild-type S. epidermidis 9142 also increased during this period (unpublished data). As a control, nematodes were transferred from one PIA-deficient S. epidermidis strain to a second PIA-deficient strain. As shown in Figure 4D, the second *ica*-deficient strain displaced the initial *ica*-deficient strain.

Taken together, the data in this section demonstrate that S. epidermidis transiently colonizes the nematode intestinal tract when transferred to an unrelated, innocuous bacterial strain. However, *icaADBC*-containing S. epidermidis has a strong competitive survival and/or growth advantage over *ica*-deficient S. epidermidis within the intestinal tract, thereby allowing PIA-producing cells to initiate a durable and ultimately fatal infection.

### S. epidermidis Biofilm Matrix in the Nematode Intestinal Tract

**Figure 5:**
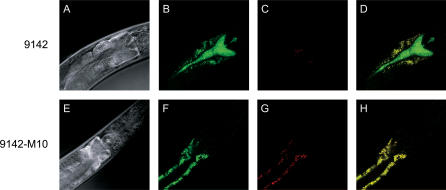
Production of PIA by S. epidermidis within the C. elegans Intestinal Tract Confocal microscopy images of C. elegans feeding on S. epidermidis labeled with FITC-conjugated WGA lectin, which selectively labels the exopolysaccharide of the biofilm matrix. Nematodes feeding on labeled wild-type S. epidermidis 9142 (A–D) or 9142-M10 (E–H). (A and E) Nomarski differential interference contrast image of anterior portion of nematodes. (B and F) Green fluorescence due to FITC-labeled lectin adhering to S. epidermidis exopolysaccharide and C. elegans intestinal autofluorescence. (C and G) Red fluorescence resulting from intestinal autofluorescence. (D and H) Merged fluorescent images in which green demonstrates bound lectin and yellow demonstrates intestinal autofluorescence.

We used fluorescein isothiocyanate (FITC)–conjugated wheat germ agglutinin (WGA) lectin to determine whether biofilm-associated polymers are present in the intestine of C. elegans feeding on *S. epidermidis.* This lectin binds to *N-*acetyl glucosamine polymers and has previously been shown to bind to the exopolysaccharide of S. epidermidis and Y. pseudotuberculosis biofilm [60,61]. Wild-type S. epidermidis 9142, but not 9142-M10, is efficiently labeled by the WGA lectin when the bacteria are grown in vitro (unpublished data). Figure 5A–5D shows that when nematodes are fed lectin-labeled wild-type *S. epidermidis,* a strong green fluorescent signal is observed in the intestinal lumen. However, nematodes feeding on similarly labeled S. epidermidis 9142-M10 lawns do not accumulate any fluorescent signal, as shown in Figure 5E–5H. Fluorescence was also observed outside of the intestinal lumen. This signal, which can be differentiated from the FITC signal by its spread into the red spectrum, is due to autofluorescence of the intestinal cells and is often enhanced in worms feeding on pathogens [62]. No fluorescent signal was observed in close approximation to the cuticle surface of the nematode. These results show that wild-type S. epidermidis 9142, but not 9142-M10, is able to produce biofilm-associated exopolysaccharide under the standard killing conditions, and that biofilm polymers are either effectively ingested by nematodes and/or that 9142 synthesizes the polymers in the C. elegans intestine.

### Overexpression of *icaADBC* Enhances S. aureus Virulence

**Figure 6:**
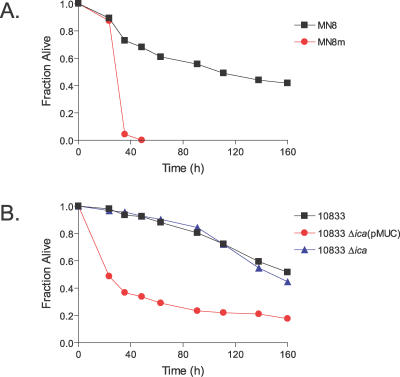
Overexpression of *icaADBC* in S. aureus Enhances Virulence (A) Survival of C. elegans N2 infected by the *icaADBC*-overexpressing S. aureus strain MN8m (circles) compared to wild-type MN8 (squares) (*p* < 0.0001). (B) Survival of C. elegans N2 infected by S. aureus strain 10833 (squares), 10833 Δ*ica* (triangles, *p* > 0.05), and by strain 10833 Δ*ica*(pMUC) (circles, *p* < 0.0001).

To further investigate the role of the *ica* locus as an independent virulence factor in staphylococci, the pathogenic behavior of S. aureus strains with altered levels of *icaADBC* expression were examined. First, we examined the behavior of the clinical strain MN8 and a spontaneously derived mutant, MN8m, which constitutively produces excess biofilm exopolysaccharide due to increased *icaADBC* transcription [17]. MN8m produces visibly mucoid lawns on TS agar plates and, as shown in Figure 6A, was dramatically more virulent towards nematodes than the parental MN8 strain (*p* < 0.0001). Despite the mucoid nature of the MN8m lawns, worms were still able to ingest the bacteria, which accumulated to high levels in the intestine similar to S. epidermidis 9142 (unpublished data).

To confirm that overexpression of the *S. aureus icaADBC* locus is sufficient to increase virulence towards *C. elegans,* we tested the effect of *icaADBC* expression in a second S. aureus strain, the clumping factor–positive strain 10833 Δ*ica* harboring the plasmid pMUC, which carries the de-repressed *icaADBC* locus from MN8m [24]. S. aureus 10833 Δ*ica*(pMUC) also produces a visibly mucoid lawn on nematode killing plates and, as shown in Figure 6B, kills nematodes much more rapidly than the parental strain 10833 (*p* < 0.0001). Interestingly, deletion of the *icaADBC* operon in 10833 or MN8 does not significantly alter the killing kinetics compared with their parental strains (Figure 6B and unpublished data), indicating that *S. aureus icaADBC* expression is likely low under the conditions used in this assay.

### PIA Production May Protect S. epidermidis in the C. elegans Intestine

**Figure 7:**
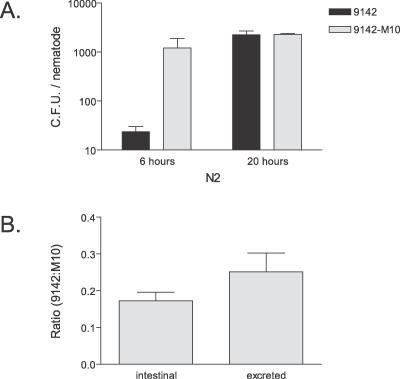
Intestinal and Fecal Ratios of Wild-Type and Biofilm-Deficient S. epidermidis in C. elegans when Fed Mixed Lawns (A) Intestinal load of S. epidermidis 9142 (dark bars) and 9142-M10 (light bars) within wild-type N2 C. elegans feeding on mixed lawns in a ratio of 1:100. Bacterial loads were determined in triplicate from approximately ten disrupted worms. Data represent mean ± SEM. (B) Ratio of S. epidermidis 9142 to 9142-M10 of excreted and intestinal bacteria. Colonized N2 C. elegans were allowed to feed on mixed lawns of 9142 and 9142-M10 (ratio 1:100) for 16 h, washed, and then transferred to M9 buffer, in which bacteria were freely excreted. Bacterial loads were determined in triplicate from the homogenates and expelled collections of approximately ten worms. Data represent mean ± SEM.

We hypothesized that staphylococcal PIA production may enhance virulence in the C. elegans model either by promoting bacterial adherence to intestinal cells and/or by increasing bacterial survival in the intestinal tract. To further investigate the mechanism by which biofilm matrix contributes to S. epidermidis virulence, a competition-based assay was performed. Wild-type N2 C. elegans were allowed to feed on lawns consisting of wild-type S. epidermidis 9142 and PIA-deficient 9142-M10 in a ratio of 1:100, respectively. Nematodes were harvested after 6 and 20 h of feeding, washed, and disrupted, and the intestinal bacterial loads of S. epidermidis 9142 and 9142-M10 quantified. As shown in Figure 7A, after 6 h of feeding, the ratio of S. epidermidis 9142 to 9142-M10 in the intestine was approximately the same as that on the feeding plates. However, after 20 h of feeding, the ratio increased to 1:1, indicating significant enrichment of wild-type 9142 compared to PIA-deficient 9142-M10 within the C. elegans intestine.

To distinguish between increased adherence and increased intestinal survival, nematodes were allowed to feed as above on a 1:100 mixed lawn for 16 h, washed, and transferred to M9 buffer, where they excreted intestinal bacteria into the media. We reasoned that if the ratio of 9142:9142-M10 was lower in the excrement than in the intestine, it would indicate that 9142 was preferentially retained compared to 9142-M10. Alternatively, if the 9142:9142-M10 ratio was equivalent in the excrement and the intestine, it would suggest that 9142 bacteria are better able to survive in the intestinal environment. As shown in Figure 7B, there was not a significant decrease in the ratio of 9142:9142-M10 in the excrement, arguing against the hypothesis that wild-type S. epidermidis is preferentially retained in the C. elegans intestine compared to PIA-deficient 9142-M10.

To determine whether PIA-producing 9142 formed focal collections of cells within the nematode intestinal tract, animals fed 9142:9142-M10 mixtures were labeled with FITC-conjugated WGA. Fluorescence appeared uniform throughout the intraluminal space, and discrete agglomerations of fluorescing cells surrounded by non-labeled bacteria were not observed. Nevertheless, we cannot exclude the possibility that such agglomerations exist in the intestinal tract and are difficult to recognize microscopically.

### Interaction of the C. elegans Innate Immune System and S. epidermidis Biofilm Production

**Figure 8:**
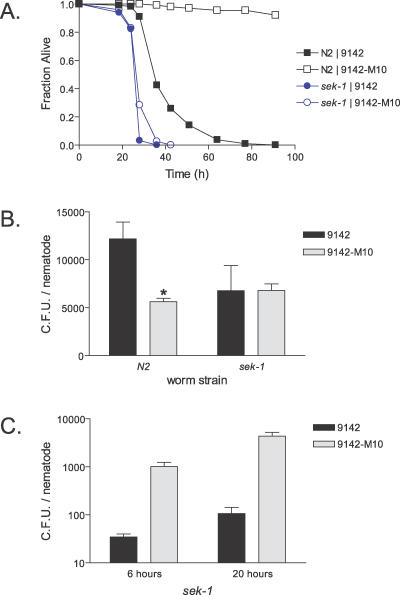
Immunocompromised C. elegans Are Hypersusceptible to both Wild-Type and Biofilm-Deficient S. epidermidis and Accumulate both Strains Equally (A) *sek-1(km4)* mutant worms (circles) are similarly susceptible to both S. epidermidis 9142 (closed symbols) and 9142-M10 (open symbols), whereas wild-type N2 nematodes (squares) are differentially resistant to 9142-M10 (*p* < 0.0001). (B) S. epidermidis 9142 (solid bars) is better able to colonize N2 worms than 9142-M10 (open bars), but there is no difference between colonization rates in *sek-1(km4)* mutant nematodes after 16 h of feeding on S. epidermidis. Data represent mean ± SEM. The asterisk indicates a significant difference (*p* < 0.05). (C) Intestinal load of S. epidermidis 9142 (dark bars) and 9142-M10 (light bars) within *sek-1(km4) C. elegans* feeding on mixed lawns in a ratio of 1:100. Bacterial loads were determined in triplicate from approximately ten disrupted worms. Data represent mean ± SEM.

In mammals, biofilm formation by pathogens plays an important role in evading the host innate immune response. To test whether S. epidermidis biofilm exopolysaccharide protects the bacteria from C. elegans immune effectors, we examined the susceptibility of C. elegans mutants deficient in innate immune response to S. epidermidis infection. As shown in Figure 2, C. elegans harboring missense mutations in the p38 MAP kinase pathway components *nsy-1* or *sek-1* were more susceptible to *S. epidermidis–*mediated killing. Similarly, the kinase domain deletion mutant *sek-1(km4)* was also more susceptible to killing by wild-type S. epidermidis (Figure 8A). Interestingly, the *sek-1(km4)* mutant was also highly susceptible to killing by the PIA-deficient S. epidermidis 9142-M10 (Figure 8A), unlike the wild-type nematode. Similarly, the C. elegans mutant *pmk-1(km25),* which contains a deletion in the gene encoding the p38-like MAP kinase downstream of SEK-1, was also highly and comparably susceptible to 9142 and 9142-M10 (unpublished data).

To determine if the sensitivity of *sek-1(km4)* nematodes to *ica*-deficient S. epidermidis–mediated killing correlated with colonization levels, the bacterial load of *sek-1(km4)* nematodes feeding on S. epidermidis 9142 and 9142-M10 for 16 h were compared to that of wild-type worms, as shown in Figure 8B. Interestingly, the bacterial load in *sek-1(km4)* nematodes is lower than in wild-type C. elegans when feeding on either 9142 or 9142-M10. However, *sek-1(km4)* animals accumulate equal intestinal loads of S. epidermidis 9142 and *ica*-deficient 9142-M10, whereas wild-type animals accumulate more 9142 than 9142-M10, as previously noted (Figure 3). The decreased accumulation of bacteria in the immunocompromised *sek-1(km4)* mutants may be a reflection of overall worm health. Indeed, *sek-1(km4)* and other immunocompromised worms were observed to perform less foraging and move more slowly through the S. epidermidis lawns compared to wild-type worms. However, the equal accumulation of wild-type S. epidermidis and PIA-deficient 9142-M10 within the intestinal tract of *sek-1(km4)* mutants correlates with their comparable nematocidal activity (Figure 8A).

If PIA production enhances the ability of S. epidermidis to colonize wild-type N2 nematodes but not immunocompromised *sek-1* mutants, then 9142 should have a competitive advantage over 9142-M10 within the nematode intestinal tract of N2, but not within *sek-1* animals during mixed infection. To test this, *sek-1(km4)* mutants were exposed to mixed lawns of 9142 and 9142-M10 in a ratio of 1:100, respectively, as was performed for N2 nematodes (Figure 7B). As shown in Figure 8C, *sek-1(km4)* mutants maintained similar proportions of 9142 and 9142-M10 at both 6 and 20 h.

To investigate whether the production of PIA by S. epidermidis may mask antigens sensed by the C. elegans p38 MAP kinase signaling pathway, worms containing green fluorescent protein (GFP) under the control of promoters of two putative PMK-1 target genes (D. H. Kim, personal communication) were exposed to wild-type and *ica*-deficient *S. epidermidis,* and no difference in GFP expression was observed (unpublished data). Consequently, we have no evidence that PIA exopolysaccharide conceals staphylococcal antigens recognized by the p38 MAP kinase signaling system, although such a function cannot be conclusively ruled out.

The data presented in this section demonstrate that, in contrast to wild-type nematodes, C. elegans with compromised innate immunity due to defective MAP kinase signaling are equally susceptible to killing by *S. epidermidis,* whether or not biofilm matrix is produced. These results support the hypothesis that biofilm exopolysaccharide in S. epidermidis protects bacteria in the C. elegans intestinal tract by modulating killing by the nematode innate immune system.

## Discussion

The best-characterized aspect of S. epidermidis virulence is its ability to form biofilm on solid surfaces, such as implanted medical devices and catheters. Indeed, the primary method for assessing S. epidermidis virulence is to measure persistence and accumulation on an implanted foreign body in a mammalian host [63]. S. epidermidis biofilm formation is not only crucial for adherence to and accumulation on artificial surfaces, but also appears to serve protective functions against antibiotics and the immune system [5,13]. As a result, biofilm-associated S. epidermidis infections of bioprosthetic materials are usually difficult to eradicate, even with prolonged antibiotic therapy, and often require removal of the colonized material in order to achieve cure.

There is now a growing body of work demonstrating the utility of using C. elegans as a model organism to study host–pathogen interactions from both the standpoints of the pathogen and the host for a variety of microbial infections (for reviews see [29] and [64]). Compared to vertebrate models, C. elegans–based models are rapid, inexpensive, and technically straightforward. In previous work, we and others have shown that S. aureus infects and kills C. elegans by a process that requires many, but not all, of the same factors necessary for full virulence in mammalian models. For example, S. aureus virulence factors or traits important for disease in both vertebrates and nematodes include virulence regulators (*sarA, saeRS,* σ^B^), exotoxins (α-toxin, V8 protease), capsule, virulence-related metabolic factors, and phenotypic variants (small colony variants) [32,34,65,66]. In contrast, adhesins of the microbial surface components recognizing the adhesive matrix molecules (MSCRAMM) family are crucial for colonization and infection in mammals but are not required for infection of nematodes [66]. Interestingly, the accessory gene regulator *(agr)* quorum-sensing system appears to contribute to S. aureus virulence in several but not all genetic backgrounds [32,66] (M. Cupp and C. Sifri, unpublished observations), which may reflect strain-dependent differences in *agr* gene regulation [67]. Here we describe the use of the C. elegans model to study the role of biofilm exopolysaccharide in S. epidermidis and S. aureus pathogenesis and host defense responses.

In this study, we demonstrated that the biofilm-associated exopolysaccharide PIA is produced by S. epidermidis when it colonizes the nematode intestinal tract. Importantly, disruption of the *icaADBC* operon, which is required for PIA biosynthesis, not only greatly diminishes the ability of S. epidermidis to infect and kill *C. elegans,* but also reduces S. epidermidis fitness during intestinal colonization. Importantly, plasmid pTX*icaADBC* complemented both biofilm formation and nematocidal activity of the *ica* mutant under xylose-inducing conditions. It should be noted that while loss of PIA production in 9142-M10 greatly diminishes virulence towards nematodes, the strain is not avirulent, as has been observed in mammalian models of S. epidermidis disease [10]. Presumably, other factors contribute to S. epidermidis virulence, a hypothesis that is supported by the high susceptibility of immunocompromised *sek-1* mutants to *ica*-deficient 9142-M10. Further experiments will be required to determine whether other putative S. epidermidis virulence factors, such as poly-γ-dl-glutamic acid, accumulation-associated protein, fibrinogen-binding protein, phenol-soluble modulins, hemolysins, extracellular proteases, or the *agr* quorum-sensing system contribute to disease in nematodes.

Biofilm formation has been previously found to play a role in Y. pestis and *Y. pseudotuberculosis*–mediated growth inhibition of C. elegans [35,36,61]. In these cases, the mechanism of virulence involves the formation of an obstructive plug over the pharyngeal opening, preventing feeding and resulting in the nematodes starving to death. Disruption of the *Yersinia hmsHFRS* locus, which is homologous to the *S. epidermidis icaADBC* locus, results in a less virulent phenotype. However, formation of an obstructive pharyngeal plug is unlikely to play a role in S. epidermidis killing, because microscopic visualization did not reveal the presence of obstructive plugs, and nematodes were able to ingest bacteria. In addition, the *C. elegans srf-2, srf-3,* and *srf-5* mutants, which are resistant to *Yersinia* infection due to the inability of the bacteria to adhere to the altered nematode cuticle, are as sensitive as wild-type animals are to S. epidermidis. Therefore, the mechanism of biofilm-mediated virulence is more likely to be a consequence of effects within the nematode intestine rather than on the surface of the nematode.

Since genetic disruption of the *S. epidermidis ica* biosynthetic locus specifically blocks PIA production and biofilm formation, it is unlikely that the lesion causes a pleiotropic phenotype. However, the lack of biofilm formation could lead to altered gene regulation and decreased virulence, since the global gene expression patterns of planktonic and biofilm bacteria differ significantly for S. aureus and several other bacterial species [68,69]. Nevertheless, the observed reduction in virulence of *S. epidermidis ica* mutants is most likely a direct consequence of reduced PIA production. This conclusion is supported by the increase in virulence observed in S. aureus strains in which the transcription of *icaADBC* is increased, as well as by the restored virulence in the pTX*icaADBC*-complemented *S. epidermidis icaA* mutant under inducing but not non-inducing conditions. It is not likely that PIA itself is toxic to nematodes, since heat-killed bacteria and culture supernatants are not harmful to *C. elegans.*

Importantly, our data suggest that wild-type S. epidermidis 9142 is better able to survive the host defense response within the C. elegans intestine than the isogenic PIA-deficient strain 9142-M10. This is reflected in the greater absolute number of bacteria recovered from worms feeding on wild-type 9142 compared to those feeding on 9142-M10, as well as in the enrichment of wild-type bacteria found within N2 C. elegans feeding on a mixed population of bacteria. In principle, this could result from greater intestinal retention of wild-type bacteria due to increased adherence (attachment hypothesis), or increased survival of wild-type bacteria in the intestinal milieu (survival hypothesis). Although we cannot definitively rule out the former possibility, we favor the survival hypothesis for the following reasons. First, nematodes feeding on mixed lawns of 9142 and 9142-M10, which are then transferred to buffer, do not excrete more PIA-deficient bacteria than wild-type bacteria. In fact, there were relatively more wild-type bacteria than PIA-deficient organisms in the expelled collections than the intestinal tract, although this difference did not reach statistical significance, suggesting that PIA-producing bacteria may have a survival advantage over PIA-deficient bacteria during transit through the digestive tract. Second, 9142 has a competitive advantage over 9142-M10 within the intestinal tract of N2 C. elegans feeding on a mixed population of 9142 and 9142-M10. By contrast, no enrichment of 9142 is observed in immunocompromised *sek-1* loss-of-function mutants feeding on a mixed population of the two strains. These results indicate that the selective advantage afforded by PIA production to 9142 during intestinal tract colonization is manifested only in animals with intact immune systems. Finally, immunocompromised *C. elegans pmk-1* and *sek-1* mutants are very sensitive to PIA-deficient *S. epidermidis,* and in *sek-1* mutants*,* in contrast to wild-type nematodes, there is no difference in bacterial titer following infection with PIA-producing and PIA-deficient S. epidermidis strains.

How SEK-1 PMK-1 p38 MAP kinase signaling promotes immunity to infection by S. epidermidis or related bacteria has yet to be determined. However, Troemel et al. recently described how the p38 MAP kinase pathway controls defense responses to Pseudomonas aeruginosa infection in C. elegans [50]. Microarray and genetic analysis show that the SEK-1 PMK-1 pathway directs a specific, inducible response to P. aeruginosa infection, characterized by activation of known or putative immune effector genes, including C-type lectins, lysozymes, neuropeptide-like proteins, homologs of ShK toxins, and proteins with CUB-like domains. If the SEK-1 PMK-1 pathway similarly regulates a specific immune response to staphylococcal infection, then the reduced production of immune effector molecules in immunocompromised *sek-1* or *pmk-1* mutants could abrogate the protective advantage of S. epidermidis biofilm exopolysaccharide. This hypothesis is consistent with the observed protection that biofilm formation confers against antibacterial peptides in vitro [70], which are believed to be among the main immune effectors utilized by C. elegans [71].

Alternatively, the SEK-1 PMK-1 pathway may regulate a nonspecific response that promotes resistance to S. epidermidis infection, and reduced stress resistance in p38 MAP kinase mutants could similarly negate the protective advantage of S. epidermidis biofilm exopolysaccharide. Furthermore, it is conceivable that disruption of the p38 MAP kinase pathway could modify the intestinal epithelium in a manner that leads to altered bacterial adherence, or that a small quantity of PIA-producing bacteria could adhere to intestinal cells, repress immune signaling, and thereby facilitate long-term colonization and increased sensitivity to killing by S. epidermidis.

If biofilm exopolysaccharide acts to impede immunological molecules as it encases an agglomeration of bacteria, then biofilm could be predicted to protect both PIA-producing bacteria and bystander bacteria. Unexpectedly, the immunoprotective action of PIA production by S. epidermidis appears to be cell autonomous within the C. elegans intestine, since wild-type S. epidermidis out-competed *icaADBC*-deficient mutants in mixed lawn feeding experiments. This result indicates that a more complex mechanism may be at play. One possibility is that the polysaccharide matrix must be modified in order to be immunoprotective and that this modification occurs in a cell-autonomous fashion. Recently, Vuong et al. showed that the *icaB* gene encodes a surface-bound enzyme, which partially deacetylates the PIA precursor. Notably, they demonstrated that deacetylation is required for resistance to the human cationic antimicrobial peptides human-β-defensin 3 and LL37 [72]. The cell autonomy of IcaB activity within the nematode intestinal tract was not investigated in this study and remains unknown.

It is interesting to speculate that the protective function of PIA represents an evolutionarily conserved activity that originated in bacteria to withstand grazing by bacterivorous nematodes and other predators. Indeed, homologs of the *ica* locus are present in a diverse array of environmental Gram-negative bacteria, including *Pseudomonas fluorescens, Xanthomonas axonopodis,* and Ralstonia solanacearum [73]. Likewise, biofilm formation has been postulated to protect environmental bacteria against predatory protozoa [74,75].

Using an interactive genetic approach, our results establish a novel in vivo experimental system for investigating the interface between staphylococcal biofilm matrix and the innate immune system. Although previous studies have investigated the contribution of biofilm production to the colonization of foreign bodies in vivo, and the immunoprotective activity of biofilm formation in vitro, we are not aware of any reports directly demonstrating the immunoprotective activity of biofilm polysaccharide in a live animal model per se. The ease of manipulation and transparency of the model system, as well as the array of genetic tools available for use in C. elegans and staphylococcal research, make the C. elegans–*Staphylococcus* infection model an attractive system for studying the interaction of biofilm formation and host defense mechanisms.

## Materials and Methods

### Strains and growth conditions.

The bacterial strains used in this study are listed in Table 1. All strains were maintained at −75 °C in TS or Luria-Bertani (LB) medium containing 15% glycerol. S. aureus strains MN8, MN8m, NCTC 10833 (herein 10833), 10833 *Δica,* and 10833 *Δica*(pMUC) were obtained from G. Pier (Channing Laboratory, Harvard Medical School, Boston, Massachusetts, United States). The sporulation-deficient B. subtilis strain RL2244 was obtained from R. Losick (Harvard University, Cambridge, Massachusetts, United States).

**Table 1:**
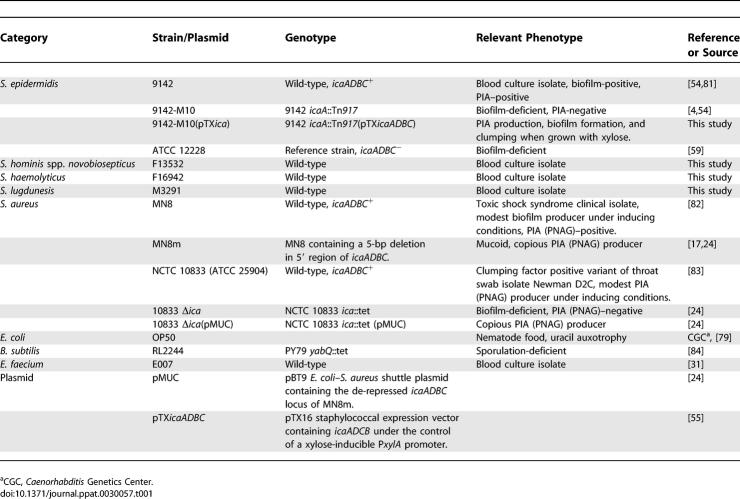
Bacterial Strains and Plasmids Used in This Study

The C. elegans strains used in this study are listed in Table 2. C. elegans strains Bristol N2, *age-1(hx546), daf-2(e1370), daf-16(mgDf47), srf-2(yj262), srf-3(yj10),* and *srf-5(ct115)* were obtained from the *Caenorhabditis* Genetics Center (http://www.cbs.umn.edu/CGC). Strain *daf-2(e1370);daf-16(mgDf47)* was obtained from D. Garsin (University of Texas, Houston, Texas, United States). Strains *sek-1(ag1), nsy-1(ag3), sek-1(km4),* and *pmk-1(km25)* have previously been described [42,76,77]. C. elegans strains were maintained at 15 °C on nematode growth medium (NGM) plates spread with E. coli strain OP50 as a food source [78,79], and were manipulated using established techniques [78].

**Table 2:**
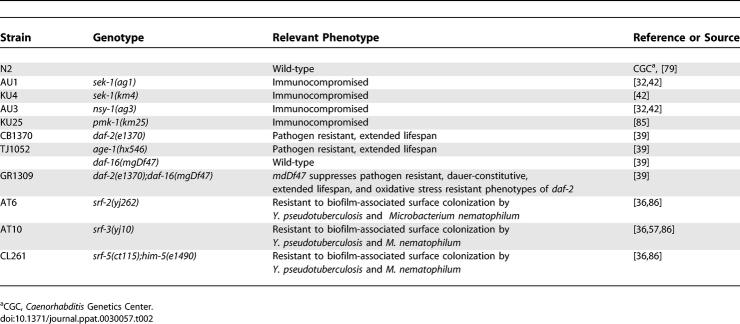
C. elegans Strains Used in This Study

### Complementation of the *ica* mutant strain.

Phage transduction of plasmid pTX*icaADBC* from S. epidermidis 9142 [55] into the *icaA*::Tn*917* mutant 9142-M10 was carried out as previously described with minor modifications [56]. In brief, phage 48, provided by V. T. Rosdahl (Staten Serum Institut, Copenhagen, Denmark), was propagated on S. epidermidis 9142(pTX*ica*), provided by F. Götz (Universität Tübingen, Tübingen, Germany), and the resulting phage lysate was used to transduce the plasmid into 9142-M10. Transductants were selected on brain heart infusion medium containing 20 μg/ml erythromycin and 20 μg/ml tetracycline.

Previous work has shown that the *icaA*::Tn*917* mutation in 9142-M10 abolishes PIA production and attachment to polystyrene [4,8]. To assess production of functional PIA, polystyrene flat bottom microtiter well adherence assays were performed as previously described using the complemented mutant S. epidermidis 9142-M10(pTX*ica*) and comparator strains grown in TS medium with or without supplemental xylose (2% wt/vol) [56].

### Nematode killing assays.

C. elegans killing assays were performed as previously described for S. aureus [32] with the following modifications. Staphylococcal strains were grown overnight at 30 °C or 37 °C with aeration in TS broth that was supplemented with 5 μg/ml nalidixic acid (Sigma, http://www.sigmaaldrich.com), 5 μg/ml tetracycline (Sigma), 10 μg/ml erythromycin (Sigma), or 5 μg/ml chloramphenicol (Sigma), as appropriate. B. subtilis was grown in LB broth containing 10 μg/ml tetracycline at 30 °C with aeration. Petri plates (3.5 cm) containing TS agar supplemented with 5 μg/ml nalidixic acid (Sigma) for staphylococcal strains were spread with 10 μl of culture and incubated at 30 °C for 6 h. These plates were allowed to equilibrate to room temperature and then were seeded with nematodes using standard techniques.

Heat-killed plates were prepared using the spotted lawn method as previously described [34], with minor modifications. Briefly, heat-killed E. coli OP50 or S. epidermidis 9142 was prepared by heating a 3-ml overnight culture to 65 °C for 30 or 60 min, respectively. Samples were streaked onto drug-free plates to ensure that the organisms were dead. The culture was pelleted by centrifugation, decanted of the supernatant, and resuspended in 300 μl of TS medium. One-hundred microliters of the concentrated heat-killed bacteria were then spotted onto TS agar plates supplemented with nalidixic acid. Plates containing live S. epidermidis and heat-killed E. coli were similarly prepared at a ratio of 1:1 or 1:5 (vol:vol).

Approximately 30 hermaphrodite nematodes in the fourth larval stage (L4) were transferred to killing plates, and their survival was monitored over time at 25 °C. Experiments were conducted in triplicate and repeated at least three times. For groups in which most nematodes survived longer than 5 d, the animals were transferred to fresh plates every 3–5 d in order to separate subjects from progeny. For experiments with live S. epidermidis heat-killed E. coli mixtures, worms were transferred to freshly prepared plates daily to ensure that heat-killed E. coli OP50 was not preferentially consumed to exhaustion during the course of the experiment. Nematodes were considered dead when they failed to respond to touch. Worms that died as a result of crawling off the plate were censored from the analysis. Nematode survival was calculated by the Kaplan–Meier method, and survival differences were tested for significance using the log-rank test (GraphPad Prism, version 4.0; GraphPad, http://www.graphpad.com). *p*-Values < 0.05 were considered statistically significant.

### Microscopic visualization of nematode intestinal tract.

Bacterial colonization of the nematode digestive tract was observed by differential interference contrast imaging with Nomarski optics using an Axioplan2 microscope (Zeiss, http://www.zeiss.com) [32].

FITC-conjugated Triticum vulgare lectin from WGA was obtained from EY Laboratories (http://www.eylabs.com) and used to fluorescently label S. epidermidis exopolysaccharide using the protocol of Tan and Darby [61] with the following modifications. S. epidermidis 9142 or 9142-M10 lawns grown for 24 h on TSA plates were scraped into 1 ml of PBS, resuspended and sonicated (three 60-second pulses of 50% duration, power level 3, on a Branson Sonifier 450) to homogenize the suspension, and washed twice with PBS to remove cell debris, with centrifugation at 16,000*g* for 3 min between washes. The cell pellet was resuspended in 1 ml of PBS containing WGA-FITC at 25 μg/ml, incubated for 60 min at room temperature with agitation, and washed three times with PBS to remove unbound WGA-FITC. Next, 50 μl aliquots of the labeled bacterial suspension were transferred to fresh Petri plates, allowed to dry, and then seeded with ten nematodes (L4) per plate. After 16 h of feeding, nematodes were examined using a Leica TCS NT confocal microscope with spectrophotometric detection by established methodologies [80].

### Quantification of nematode colonization and excretion.

For quantification of bacterial colonization of C. elegans and rates of differential excretion, nematodes were allowed to feed on S. epidermidis strain 9142, 9142-M10, or a mixture of both under standard killing conditions. Approximately 30 nematodes were transferred manually from the killing plates into a 250-μl drop of M9 buffer, divided into three pools, and washed three times with 200 μl of M9 buffer containing 1 mM sodium azide by serial transfer in a 96-well micro titer plate using a Pasteur pipette. Nematodes were then transferred into 2-ml conical tubes, and the volume was increased to 250 μl with fresh M9 buffer without added sodium azide. Worms were homogenized by adding approximately 200 μl of sodium carbide beads (BioSpec Products, http://www.biospec.com) and vortexing for 1 min. Serial dilutions of the supernatant were made to determine the number of viable bacteria.

To evaluate persistent bacterial colonization of *C. elegans,* nematodes were allowed to feed on S. epidermidis strain 9142, 9142-M10, or ATCC 12228 under standard conditions. After 12 h of feeding on the initial (pulse) S. epidermidis strain, worms were transferred to a second (chase) S. epidermidis strain. Prior to and 20 and 40 h after transfer, approximately 30 nematodes were transferred, washed, homogenized, and plated as described above. Serial dilutions of the homogenates of nematodes transferred from S. epidermidis 9142 to 9142-M10 or ATCC 12228 were plated on TS agar plates containing 0.2% Congo Red and 0.25% additional glucose (CRA_TS_), and the plates were incubated overnight at 37 °C. Under these conditions, wild-type S. epidermidis 9142 appeared black, whereas the *ica* mutant strain 9142-M10 and the *ica*-deficient strain ATCC 12228 appeared red [16]. Similarly, serial dilutions of the homogenates of nematodes transferred from 9142-M10 (erythromycin resistant) to ATCC 12228 (erythromycin sensitive), or vice versa, were replica plated on TS agar plates with and without 10 μg/ml erythromycin for enumeration. The Fitness Index is calculated as follows: (pulse S. epidermidis strain C.F.U) / (pulse S. epidermidis strain C.F.U. + chase S. epidermidis strain C.F.U), where the pulse strain is the initial S. epidermidis strain, and the chase strain is the second S. epidermidis strain.

To determine excretion rates, three groups of ten nematodes each were transferred to 1.5-ml tubes containing 500 μl of M9 buffer without sodium azide. After 2 h, the number of C.F.U. were determined from aliquots of the solution and worm homogenates by plating serial dilutions on CRA_TS_. Differences in quantified intestinal tract and/or excreted bacteria were compared for statistical significance using a standard two-tailed *t*-test (GraphPad Prism, version 4.0). *p*-Values < 0.05 were considered statistically significant.

## Supporting Information

Figure S1 Coagulase-Negative Staphylococcal Species other than S. epidermidis Kill C. elegans
Survival of N2 C. elegans on strains F13532 (Staphylococcus hominis spp. *novobiosepticus,* squares), F16942 (*Staphylococcus haemolyticus,* triangles), and M3291 (*Staphylococcus lugdunensis,* diamonds) compared to ATCC 12228 (biofilm-deficient nonpathogenic S. epidermidis reference strain, circles).
(1.5 MB PDF)

## Abbreviations

C.F.U.: colony-forming unit
FITC: fluorescein isothiocyanate
*ica,*: intercellular adhesion locus
MAP: mitogen-activated protein
PIA: polysaccharide intercellular adhesin
PNAG: β-1,6-linked polymeric *N*-acetyl glucosamine
SEM: standard error of the mean
TS: tryptic soy
WGA: wheat germ agglutinin
