# Supplementary material for: Staphylococcal Biofilm Exopolysaccharide Protects against Caenorhabditis elegans Immune Defenses
Source: PLoS Pathog. 2007 Apr 20;3(4):e57. doi: 10.1371/journal.ppat.0030057 (PMC1853117; doi:10.1371/journal.ppat.0030057)
Supplement: Supplementary file 1 [file ppat.0030057.sg001.pdf]

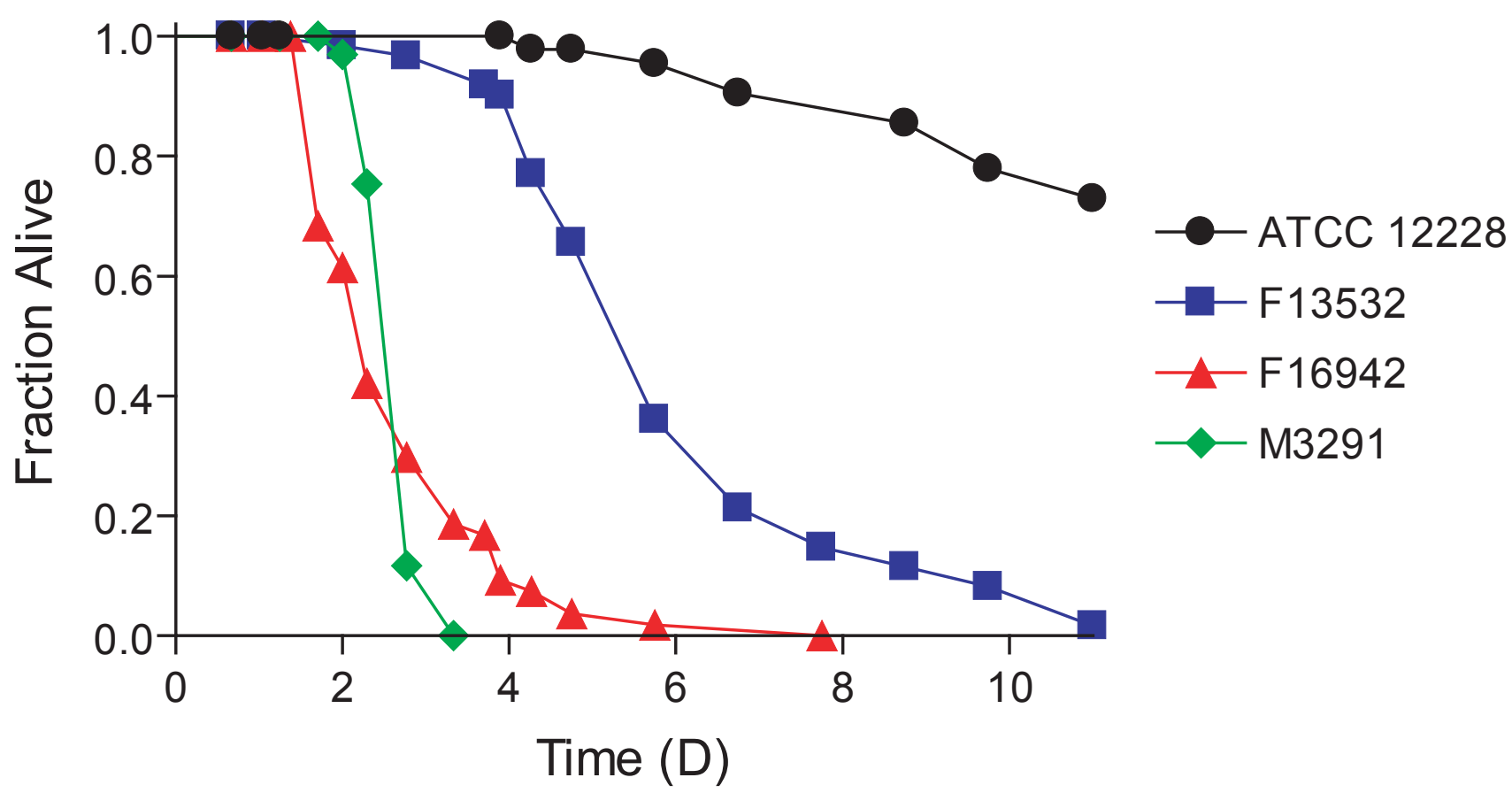

Fraction Alive
1.0
0.8
0.6
0.4
0.2
0.0
0
2
4
6
8
10
Time (D)
ATCC 12228
F13532
F16942
M3291
